# Evolution of genetic architecture and gene regulation in biphenyl/PCB-degrading bacteria

**DOI:** 10.3389/fmicb.2023.1168246

**Published:** 2023-06-07

**Authors:** Hidehiko Fujihara, Jun Hirose, Hikaru Suenaga

**Affiliations:** ^1^Department of Food and Fermentation Sciences, Faculty of Food and Nutrition Sciences, Beppu University, Beppu, Japan; ^2^Department of Applied Chemistry, Faculty of Engineering, University of Miyazaki, Miyazaki, Japan; ^3^Cellular and Molecular Biotechnology Research Institute, National Institute of Advanced Industrial Science and Technology (AIST), Tokyo, Japan

**Keywords:** xenobiotic compounds, degrading bacteria, mobile genetic elements, gene regulation and expression, adaptive evolution

## Abstract

A variety of bacteria in the environment can utilize xenobiotic compounds as a source of carbon and energy. The bacterial strains degrading xenobiotics are suitable models to investigate the adaptation and evolutionary processes of bacteria because they appear to have emerged relatively soon after the release of these compounds into the natural environment. Analyses of bacterial genome sequences indicate that horizontal gene transfer (HGT) is the most important contributor to the bacterial evolution of genetic architecture. Further, host bacteria that can use energy effectively by controlling the expression of organized gene clusters involved in xenobiotic degradation will have a survival advantage in harsh xenobiotic-rich environments. In this review, we summarize the current understanding of evolutionary mechanisms operative in bacteria, with a focus on biphenyl/PCB-degrading bacteria. We then discuss metagenomic approaches that are useful for such investigation.

## Introduction

Xenobiotic compounds are man-made chemicals that are present at unnaturally high concentrations in the natural environment. A variety of bacteria in the environment can utilize various xenobiotic compounds as a source of carbon and energy (Van der Meer et al., 1992). Phylogenetically unrelated bacterial strains often share similar metabolic pathways and enzyme systems for the degradation of xenobiotic compounds. It is believed that bacteria have acquired the ability to degrade even xenobiotic compounds they have never encountered (Nagata et al., 2015; Jeffries et al., 2018; Miglani et al., 2022). The bacterial strains degrading xenobiotics are suitable models to investigate the adaptation and evolutionary processes of bacteria because they appear to have emerged relatively soon after the release of these compounds into the natural environment.

Polychlorinated biphenyls (PCBs) are xenobiotic compounds in which the aromatic biphenyl carbon skeleton contains between one and 10 chlorine atoms. The high chemical stability, superhydrophobicity, and toxicity of PCBs make them some of the most serious and persistent environmental pollutants (Abraham et al., 2002; Furukawa and Fujihara, 2008). It is therefore somewhat surprising that many microbes that are capable of degrading PCBs have been identified. A number of biphenyl-utilizing bacteria with the ability to degrade PCBs have been isolated and characterized (Pieper and Seeger, 2008). Lignin is a complex substance with a phenylpropane structure; it contains various biphenyl molecules and is widely distributed throughout the earth. Biphenyl-degrading bacteria are thought to be responsible for the final stage of lignin degradation (Iram et al., 2021). Since biphenyl dioxygenase, the first enzyme in the biphenyl catabolic pathway, hydroxylates plant-derived flavonoids (Zubrova et al., 2021) and its homologous enzyme oxidizes dehydroabietic acid (Witzig et al., 2007), a biphenyl-degrading pathway might be involved in the degradation of plant secondary metabolites other than lignin. Thus, the ancestry of biphenyl-utilizing bacteria and their catabolic genes is quite ancient, and the genes may be distributed across a wide range of bacteria. Further, they would have the potential to adapt to different aromatic compounds, including PCBs. Therefore, the biphenyl/PCB-degradation system in bacteria appear to be a suitable model for the study of microbial adaptive evolution.

Recently, many bacterial genomes and metagenomes derived from environments contaminated with xenobiotic compounds have been analyzed at an accelerated pace (Garrido-Sanz et al., 2018; Hirose et al., 2019; Miglani et al., 2022). Much evidence has been found to support the idea that different biphenyl/PCB-degrading bacteria have evolved in the environment through different processes. In this review manuscript, we summarize the diversity, recruitment, and expression of degradation genes for biphenyl/PCB, shedding light on the sophistication of degradation gene systems and the adaptive evolution of these host bacteria.

### Bacterial mobile genetic elements

Mobilization of the catabolic genes in bacteria can be accomplished through a variety of mobile genetic elements (MGEs), including plasmid, transposon, and integrative and conjugative elements (ICEs). These genes are modified and rearranged in different ways in host bacterial cells. Degradation gene clusters for biphenyl/PCB (*bph*) are often located on MGEs, and can be transferred between bacterial cells, conferring degradation capacity to non-degrading bacteria (Van der Meer et al., 1992; Tsuda et al., 1999; Springael and Top, 2004; Satola et al., 2013).

#### Plasmids

Plasmids are mobile genetic elements that facilitate rapid adaptation and evolution by conjugative transfer between bacterial cells in the environment (Smillie et al., 2010; Aminov, 2011; Rodríguez-Beltrán et al., 2020). Catabolic plasmids contain the complete set of genes encoding the enzymes for the degradation of a xenobiotic compound. They are relatively large (more than 50 kb), due to the presence of numerous insertion sequences (ISs) and transposons. The important characteristic of catabolic plasmids is incompatibility. That is, plasmids are classified into incompatibility (Inc) groups based on their replication and partitioning systems; two plasmids of the same group cannot replicate in the same cell and are considered incompatible (Top and Springael, 2003; Popowska and Krawczyk-Balska, 2013; Shintani et al., 2015). Genes for xenobiotic degradation are often found on broad-host-range IncP-1 plasmids, such as pSS60 (Burlage et al., 1990), pBRC60 (Burlage et al., 1990) and pJP4 (Newby et al., 2000).

A variety of PCB-degrading phenotypes have also been attributed to catabolic plasmids (Furukawa and Fujihara, 2008). The *bph* genes of thermophilic *Geobacillus* sp. strain JF8 are located on a plasmid pBt40 (Mukerjee-dhar et al., 2005). *Rhodococcus* sp. RHA1 harbors large linear plasmids, including pRHL1 (1,100 kb), pRHL2 (450 kb), and pRHL3 (330 kb), and its *bph* genes are mainly located on the pRHL1 (Shimizu et al., 2001). Many other *bph* gene clusters have been identified on the linear mega-plasmids of different *Rhodococcus* species (Taguchi et al., 2004; Garrido-Sanz et al., 2020). Both the order and sequence of the *bph* genes have been shown to differ among rhodococci, and there is evidence of recombination around *bph* gene clusters, such as insertion of transpose (Taguchi et al., 2007). These findings suggest that these *bph* gene clusters evolved separately and were spread in rhodococci by horizontal transfer. *Cupriavidus* sp. SK-4, a PCB-degrading strain reported to utilize di-*ortho*-substituted biphenyl, was found to harbor a single plasmid: pSK4. Experimental results showed that pSK4 could be mobilized into *Pseudomonas putida* MB1335 and the PCB-degrading enzymes could be expressed in this strain (Ilori et al., 2015). Bacterial plasmids are important vehicles for horizontal gene transfer (Redondo-Salvo et al., 2020), and can therefore play a key role in the evolution of catabolic pathways and their hosts.

#### Transposons

Transposons are defined segments of DNA that are able to move from one genetic location to another target location in the absence of any nucleotide sequence homology. Most bacterial transposable elements, including ISs and transposons, can be traditionally classified into three classes: class I, class II, and conjugative transposons (Tan, 1999; Tsuda et al., 1999). However, a revised classification system has been proposed wherein conjugative transposons, genomic islands, and integrative plasmids would be collectively called ICEs (Burrus and Waldor, 2004; Johnson and Grossman, 2015).

Bacterial class I elements include the simple ISs, which carry only the genetic determinants for transposition (usually transposase) and composite transposons, in which various genetic traits unrelated to transposition are flanked by two copies of very similar ISs in direct or inverted orientation (Ross et al., 2021). Bacterial class II transposons generally carry the genes for their transposition (transposase, and resolvase) and one or more phenotypic traits between their terminal inverted repeats (Ross et al., 2021). It has been reported that several bacterial class II transposons play a crucial role in the widespread distribution of various catabolic gene clusters, such as the cluster of genes encoding toluene, naphthalene, and carbazole degradation pathways (Tsuda et al., 1999; Sota et al., 2006; Nojiri, 2012). Only a few bacterial transposons carrying catabolic genes for biphenyl/PCB have been experimentally proven to be mobile. An example of a functional catabolic transposon is Tn*5280*, which was identified on plasmid pP51 of chlorobenzene-utilizing *Pseudomonas* sp. strain P51 (Tan, 1999; Top et al., 2002). Tn*5280* transposes randomly at different chromosomal sites in *P. putida* KT2442 (van der Meer et al., 1991). This strongly suggests that some of the PCB degradation genes in strain P51 originated from a toluene or benzene degradation pathway, probably by HGT.

#### Integrative and conjugative elements

**Figure 1:**
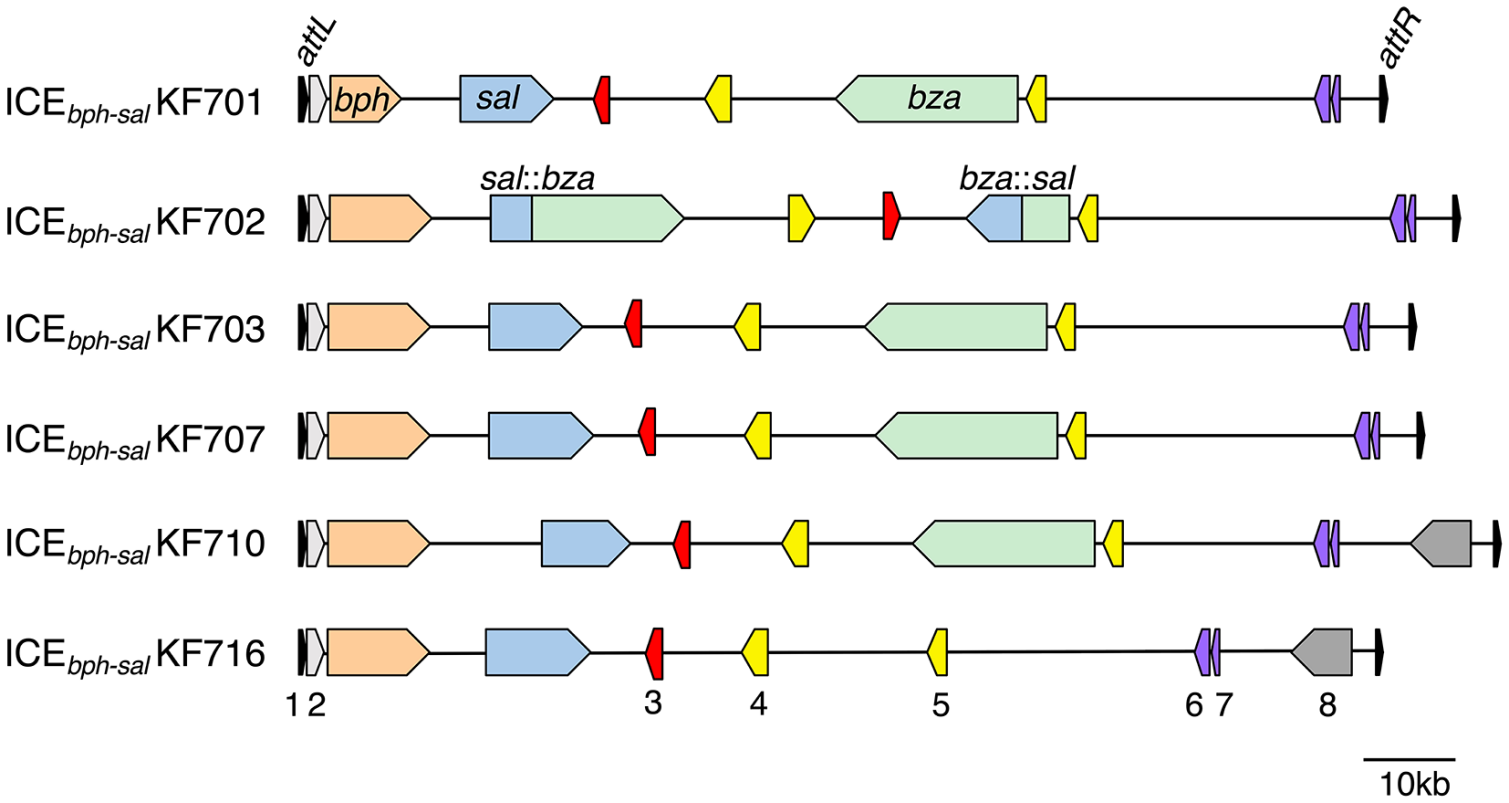
Organization of the ICE*_bph-sal_* in KF strains. ICE*_bph-sal_*KF701, ICE*_bph-sal_*KF702, ICE*_bph-sal_*KF703, ICE*_bph-sal_*KF707, and ICE*_bph-sal_*KF710 carry the *int* gene, *bph* genes, *sal* genes, and *bza* genes. ICE*_bph-sal_*KF716 carries the *int* gene, *bph*, and *sal* genes, but not the *bza* genes. The *bza* genes and *sal* genes in ICE*_bph-sal_*KF702 are formed in a fusion gene. 1, tRNA-Gly (CCC) genes (partial) (black); 2, *int* genes (grey); 3, *traI* genes (red); 4, VirB4 components of the type IV secretory pathway (yellow); 5, a VirD4 component of the type IV secretory pathway (yellow); 6, *parB* genes (purple); 7, *parA* genes (purple); and 8, YbhFSR ABC-type transporter (dark grey). The *attL* (18 bp) site, including the 18 bp of the 3′ end of the tRNA-Gly gene, and *attR* (18 bp) site are indicated.

Integrative and conjugative elements (ICEs) are bacterial mobile genetic elements that are excised from the chromosome by site-specific recombination between the attachment ends (*attL* and *attR*) mediated by an ICE-specific integrase (Figure 1; Delavat et al., 2017). The excised ICE molecule undergoes single-strand cleavage at the origin of transfer (*oriT*). TraI (relaxase) binds to the *oriT* of the circular intermediate of the ICE, cleaves one strand and binds to the 5′ end, which is then recognized by the VirD4 complex and transported through the mating pair formation system (MPF) to a recipient cell. VirD4 and VirB4 are large (> 70 kDa) ATPase proteins, and VirB4 is a component of MPF (Christie et al., 2014). The double-stranded DNA is reconstituted and site-specifically recombines with the recipient’s *attB* attachment site on the chromosome to become re-integrated (Guglielmini et al., 2011). The integration sites in the genome of the recipient cell, such as the structural genes for tRNAs, have been reported for many conjugative transposons (Wozniak and Waldor, 2010; Delavat et al., 2017).

Although ICEs often carry cargo genes involved in pathogenicity, antibiotic resistance and heavy metal resistance to endow hosts with phenotypes beneficial for niche adaptation (Bellanger et al., 2014), they are also known to carry cargo genes for the catabolism of xenobiotic compounds (Hirose, 2023). ICE*clc* is a well-known ICE in xenobiotic biodegradation and carries cargo genes involved in the metabolism of chlorocatechol (*clc* genes) and aminophenol. The element was originally identified in the 3-chlorobenzoate-degrader *Pseudomonas knackmussii* B13 (Gaillard et al., 2006), and was almost identical to the ICEs inserted into the chromosome of *Paraburkholderia xenovorans* (formerly *Burkholderia xenovorans*) LB400 (ICE*clc*-LB400) (Chain et al., 2006) and *P. aeruginosa* JB2 (ICE*clc*-JB2) (Obi et al., 2018). *P*. *xenovorans* LB400 is also a well-characterized PCB-degrading bacteria, in which chlorobenzoate is formed during the degradation of PCB (Mondello, 1989). This bacterial strain provide insight into the roles of ICEs in the evolution of catabolic pathways for the biodegradation of chlorinated aromatic compounds. On degradative ICEs, catabolic gene products (e.g., *bph*, *nah*, and *sal*) that are related to the same substrate always share nearly 100% identity, whereas the sequences of the gene products of the transmission module exhibit variations (Mohapatra et al., 2022; Hirose, 2023). Although it has been suggested that there is a mechanism governing catabolic gene insertion and exchange on ICEs, there is little evidence to support this idea.

It is known that some biphenyl/PCB catabolic genes, *bph,* are horizontally transferred via ICEs. Several of the degradative ICEs carrying the *bph* gene belong to either of two groups, the Tn*4371* family or ICE*clc* family, whose respective members share a common core region. The transfer module is required for the conjugal transfer from donor to recipient forming the type IV secretion system (T4SS) (Bellanger et al., 2014; Delavat et al., 2017). Many components required for conjugal transfer constitute a “core region” that is conserved among ICE family members. Tn*4371* is the first ICE carrying *bph* genes and was found in the chromosome of *Cupriavidus oxalacticus* A5 (Springael et al., 1993). The chromosome of *Acidovorax* sp. strain KKS102 contains ICE_KKS102_*4677* (Ohtsubo et al., 2012), which belongs to the Tn*4371* family (Toussaint et al., 2003). ICE_KKS102_*4677* is known to be transferred by conjugation to a wide range of bacteria across the genera via a circular intermediate. The *bph* genes of *Cupriavidus basilensis* KF708 and *Commamonas testosteroni* KF712 are also located on an ICE. The genes ICE*_bph_*KF708 and ICE*_bph_*KF712, which carry the *bph* genes of KF708 (Suenaga et al., 2015) and KF712 (Hirose et al., 2015a), are almost identical to ICE_KKS102_*4677* and Tn*4371,* respectively. A new ICE*clc* family carrying *bph* genes and salicylic acid catabolic genes, *sal,* was found in the PCB-degrading strain *Pseudomonas stutzeri* KF716 (Hirose et al., 2015b). The ICE*_bph-sal_*KF716 contains common core regions that show homology with those of ICE*clc* from *P. knackmussii* B13 (Gaillard et al., 2006) and ICE*_XTD_* from *Azoarcus* sp. CIB (Zamarro et al., 2016). A comparison of the genetic loci revealed that several putative ICEs from *P. putida* B6-2 (Tang et al., 2011), *P. alcaliphila* JAB1 (Ridl et al., 2018), *P. stutzeri* AN10 (Brunet-Galmés et al., 2012), and *P. stutzeri* 2A20 (Heinaru et al., 2016) had core regions highly conserved with those of ICE*_bph-sal_*KF716, along with a variable region encoding the catabolic genes for phenol, naphthalene and biphenyl. ICE*_bph-sal_*KF716 was reported to have been transferred from *P. stutzeri* KF716 to *P. aeruginosa* PAO1 via a circular extrachromosomal intermediate form (Hirose et al., 2021). These reports demonstrate that ICE subfamily members that share core regions highly conserved with those of ICE*bph-sal*KF716 are widely distributed among aromatic-degrading bacteria.

### Evidence of the evolution of PCB-degradation gene clusters in polluted sites

In the past few years, several novel studies have provided insights into the diversity and evolution of biphenyl/PCB catabolic genes in the process of adaptation to environmental niches (Hirose et al., 2019, 2021). Furukawa et al. determined the whole genomes of 10 biphenyl/PCB-degrading bacterial strains (KF strains) isolated from a biphenyl-contaminated soil sample (Furukawa et al., 1989). Genome analyses revealed that all 10 strains had the *bph* genes, while seven strains also had *sal* genes (Figure 1). A series of ICEs named ICE*_bph-sals_* that were larger (more than 110 kb) than many other ICEs (Bellanger et al., 2014) contained highly conserved *bph* genes and *sal* genes (Hirose et al., 2019). Most of these ICE*_bph-sals_* possessed benzoate catabolic genes encoding the extradiol cleavage (*bza*) pathway. The fusion gene cluster of *sal:bza* and *bza:sal* was found in ICE*_bph-sal_*KF702 (Fujihara et al., 2015), and was likely generated by homologous recombination between the *sal* and the *bza* genes.

ICE*_bph-sal_* of *P. putida* KF715 existed both in an extrachromosomal circular form (referred to as ICE*_bph-sal_* [circular] or pKF715A; hereinafter called pKF715A) and an integrated form in the chromosome (referred to as ICE*_bph-sal_*KF715 [integrated]) in stationary phase culture (Suenaga et al., 2017). The ICE*_bph-sal_* KF715 was transferred at high frequency into *P. putida* AC30 and *P. putida* KT2440, and it was stably maintained in a circular form, pKF715A. The pKF715A in these transconjugant strains was further transferred into *P. putida* F39/D, and it existed in an integrated form in the chromosome. The structural features of *bph* and its flanking regions between KF701 and KF715 were almost identical, indicating that the *bph-sal* clusters were horizontally transferred to one another at this site between KF701 and KF715. Various mobile genes encoding transposases and a retron encoding retron-type reverse transcriptases have been shown to be inserted in the *bph-sal* clusters of ICE*bph-sal*s. It has been hypothesized that these inserted sequences contribute to the exchange of the *bph* gene with the upper *nah* operon, the counterpart of the *bph* gene in the *nah-sal* cluster which is involved in naphthalene catabolism (Hirose et al., 2021; Mohapatra et al., 2022). These studies demonstrate that the *bph* gene clusters have integrative functions, are transferred among soil bacteria by various MGEs, including plasmids, transposons, and ICEs, and are diversified through modification.

### Regulation of catabolic operons

**Figure 2:**
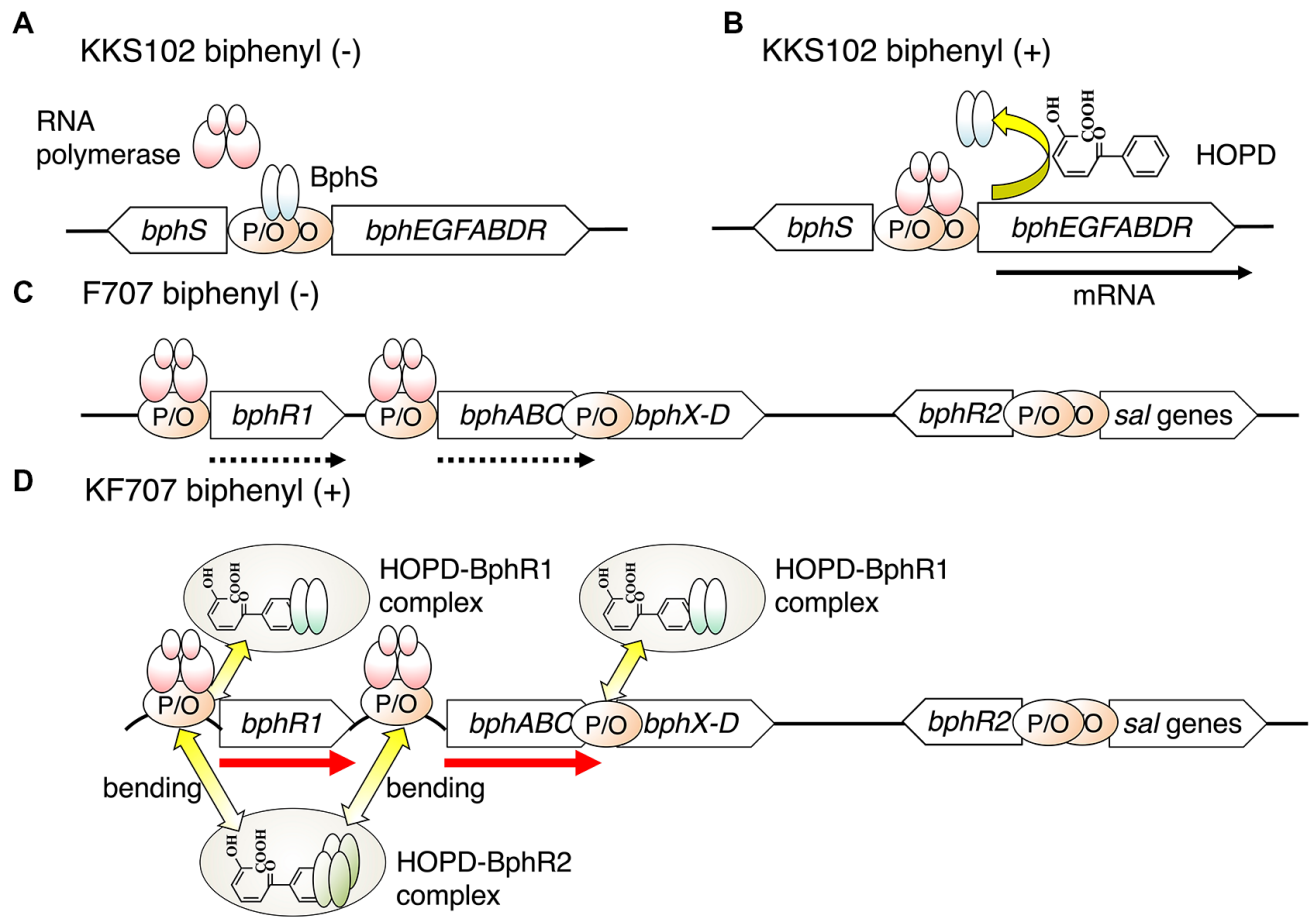
A transcriptional model of the *bph* gens in KKS102 in the absence **(A)** and in the presence **(B)** of biphenyl, and in KF707 in the absence **(C)** and in the presence of biphenyl **(D)**. The black arrow shows induced transcription, the dotted arrow constitutive transcription, and the red arrow upregulated transcription. P, promoter; O, operator.

The regulatory systems of aromatic compound-degradation genes are crucial evidence of the evolution of xenobiotic compound-degradation genes such as biphenyl, naphthalene, and phenol. In general, the expression of these degradation genes is controlled by one or more regulatory proteins (Diaz and Prieto, 2000). The regulatory protein binds to the effector, the substrate itself, or intermediates, thereby affecting the transcription of the operons. The many PCB catabolic operons in Gram-negative bacteria are regulated by regulatory proteins belonging to the GntR family (Merlin et al., 1997; Watanabe et al., 2000; Ohtsubo et al., 2001). In general, the regulators belonging to the GntR family acts as repressors (Tropel and van der Meer, 2004). The regulators bind to the operator region in the absence of substrates (biphenyl/PCBs) and inhibit the transcription of their operon (Figure 2). One such strain, KKS102, possesses a *bph* operon consisting of *bphSEFGA1A2A3BCA4R.* This operon is downregulated by a BphS belonging to the GntR family in the absence of HOPD (2-hydroxy-6-oxo-6-phenylhexa-2,4-dienoic acid), an intermediate of biphenyl catabolism (Ohtsubo et al., 2001). The *bph* genes in Tn*4371* isolated from *C. oxalacticus* A5 consisting of *bphEFGA1A2A3BCD* are also downregulated by BphS belonging to the GntR family (Merlin et al., 1997). These degrading microorganisms are thought to reduce energy consumption by suppressing the transcription of degradation genes in the absence of substrates.

On the other hand, in *Pseudomonas furukawaii* KF707 (formerly *P. pseudoalcaligenes* KF707) (Kimura et al., 2018), a *bph* gene cluster consisting of *bphR1A1A2A3A4BCX0X1X2X3D* is upregulated by BphR1 (Watanabe et al., 2000). This regulator protein also belongs to the GntR family and acts as an activator having an effect opposite that of KKS102 (Ohtsubo et al., 2001). The details of this regulator protein will be described later. In addition, *bph* genes in *P. xenovorans* LB400 are also upregulated by ORF0, which belongs to the GntR family (Denef et al., 2004). This phenomenon is likely caused by the positional relationship of the promoter and operator of *bph* genes (Figure 2). In strain KKS102, the promoter and operator sequence of *bphS* exists between the *bphS* and *bphEGFABDR* genes, and thus the binding of BphS on the operator inhibits the binding of RNA polymerase on the promoter sequence of the *bphEGFABDR* genes (Ohtsubo et al., 2001). On the other hand, in strain KF707, the promoter and operator regions of the *bphR1* and *bphA* genes do not overlap, and thus the binding of BphR1 on the *bphR1* operator does not inhibit the binding of RNA polymerase. The binding of a complex of HOPD-BphR1 and HOPD-BphR2 on the operator could induce the bending of DNA molecules and enhance the binding of RNA polymerase on the promoter region of *bph* genes (Figure 2). Therefore, the expression of the *bph* operon is greatly enhanced in the presence of biphenyl in strain KF707 (Watanabe et al., 2000, 2003). Although the regulatory system for biphenyl/PCB degradation differs between strains KKS102 and KF707, in both cases the regulatory gene plays an important role in the efficient use of energy for growth and in the survival of the host in polluted environments.

The KF707 strain has another putative regulator, BphR2, that belongs to the LysR family and acts as an activator of the *bph* upper operon (*bphR1* and *bphABC*) (Figure 2). The *bphR2* gene is located far downstream (6.6 kb) of the *bph* genes and just upstream of the salicylate catabolic (*sal*) genes (Fujihara et al., 2006). BphR2 binds to the operator regions of *bphR1* and *bphA1* and activates the transcription of the *bph* upper operon. Interestingly, BphR2 also activates the transcription of *sal* genes in the presence of hydroxy muconate semi-aldehyde, the intermediate of salicylate. This regulatory behavior is the same as that of NahR, the regulator of the naphthalene catabolic (*nah*) operon. Actually, the *bphR2* and *sal* genes of strain KF707 have high similarity to the *sal* genes of the *nah* gene cluster containing *nahR* on *P. stutzeri* AN10 (Bosch et al., 1999). This indicates that the *bph-sal* gene cluster of strain KF707 might have evolved through module exchange between the *nah* gene and *bph* gene in the *nah-sal* cluster.

Several investigations into the regulation of biphenyl/PCB-degrading Gram-positive bacteria have also been reported. In strain *R. jostii*, RHA1 possesses diverse biphenyl/PCB-degradation genes that encode multiple isozymes for each metabolism step and are distributed among multiple clusters (Takeda et al., 2004). The transcriptions of these genes are induced by dual regulatory systems. In the presence of biphenyl, a BphS1T1 two-component system induced five biphenyl-degrading gene clusters. And another two-component system, BphS2T2, also induced the biphenyl-degrading genes. However, in these cases the effector molecule was not biphenyl but other aromatic compounds such as ethylbenzene, benzene and so on (Takeda et al., 2010). And the transcriptions of *bpd* genes in another biphenyl/PCB-degrading gram-positive bacteria, *Rhodococcus* sp. strain M5, are regulated by a BpdST two-component system that is induced by the biphenyl (Labbé et al., 1997).

### Control of the gene expression

In order to survive under adverse conditions, bacteria transitorily induce the expression of particular genes to deal with environmental stresses. Tandem repeats (TRs) of short nucleotide sequences are often found in the intergenic regions in bacterial genomes (van Belkum et al., 1998). Several studies have reported that the rearrangement of TRs is involved in the gene expression of bacteria at either the transcriptional or translational level (Gemayel et al., 2010; Zhou et al., 2014).

**Figure 3:**
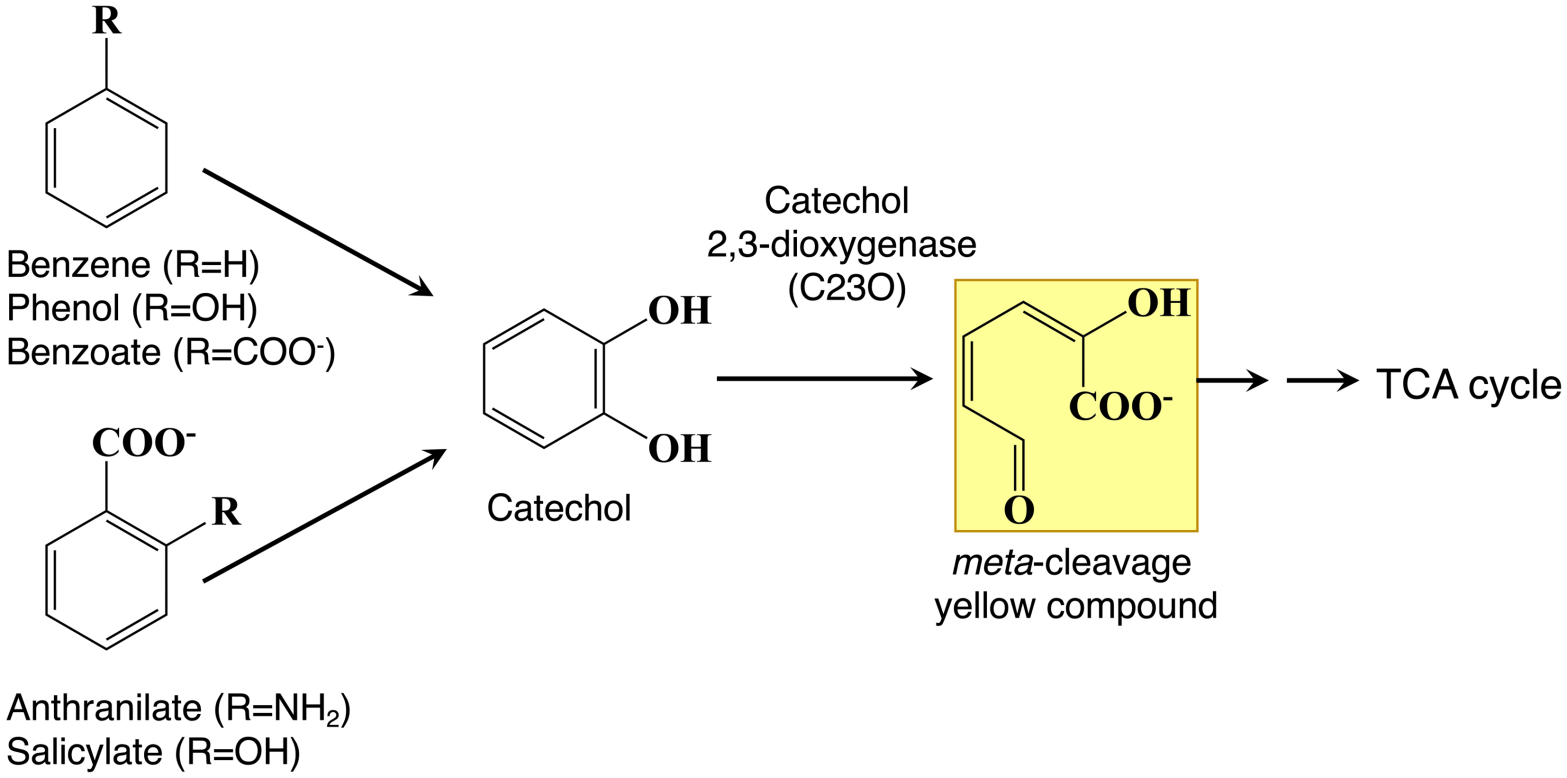
Bacterial degradation pathways of aromatic compounds. Aromatics are converted to catechol as a central intermediate. Catechol is cleaved by catechol 2,3-dioxygenase (C23O) to produce *meta*-cleavage yellow compounds, ultimately leading to TCA cycle intermediates.

Recently, a novel TR sequence, T(G/A)ACATG(A/C)T, and polymorphisms consisting of a number of repeats located in the region upstream of catechol 2,3-dioxygenase (C23O)-encoding genes were identified by metagenomic analysis (Suenaga et al., 2022). The level of protein expression of C23O dramatically increased as the number of TRs increased, reaching a maximum value with three and four repeats. Experimental results indicated that this nonanucleotide TR would affect the translational efficiency of the gene expression system. A metagenomic sample was collected from activated sludge used to treat industrial wastewater that contained mono- and polycyclic aromatic chemicals (Suenaga et al., 2009). C23O is a key enzyme in the degradation of aromatic compounds because catecholic compounds are the common intermediates in the degradation pathways (Figure 3; Hirose et al., 1994). In this harsh, aromatic-rich environment, the increase in C23O activity realized by adjusting the number of TRs is probably providing the host microbes a survival advantage. In fact, a metagenomic analysis demonstrated that, among the polymorphisms consisting of a number of repeats, tandem repeats of three and four, which indicate higher C23O enzyme activity, dominate in this environment (Suenaga et al., 2022).

## Future perspective

In this review, based on the genome sequence of isolated xenobiotic compound- degrading bacteria, the current understanding of the evolutionary mechanisms occurring in degrading gene systems is presented. DNAs of particular interest, such as MGE, within the genomes of these bacterial strains have been a focus of recent studies and have been carefully analyzed. The selective collection of DNA molecules from the environment also seems to be efficient for the study of these evolutionary mechanisms. For instance, plasmids are a relatively undiscovered region of the genome and frequently contain genes that are essential for the survival of the host, such as genes involved in biodegradation and antibiotic resistance. Therefore, a metaplasmid or metamobilome study targeting total plasmid DNA in the environment is a more effective approach for understanding the content and composition of genes in microbial communities for key ecological processes (Suenaga, 2012; Alanin et al., 2021). The comprehensive acquisition of degradative genes by a metaplasmid approach at a single site will contribute to our understanding of their on-site adaptive evolution.

## Author contributions

HS drafted the original manuscript. All authors critically reviewed and revised the manuscript draft and approved the final version for submission.

## Funding

This work was performed as part of a project supported by JSPS KAKENHI and the Institution for Fermentation, Osaka (IFO).

## Conflict of interest

The authors declare that the research was conducted in the absence of any commercial or financial relationships that could be construed as a potential conflict of interest.

## Publisher’s note

All claims expressed in this article are solely those of the authors and do not necessarily represent those of their affiliated organizations, or those of the publisher, the editors and the reviewers. Any product that may be evaluated in this article, or claim that may be made by its manufacturer, is not guaranteed or endorsed by the publisher.
